# Tumor vasculature: a sally port for inhibiting cancer cell spreading

**DOI:** 10.1186/s40880-018-0322-z

**Published:** 2018-08-03

**Authors:** Chao-Nan Qian, Francesco Pezzella

**Affiliations:** 1 State Key Laboratory of Oncology in South China, Collaborative Innovation Center for Cancer Medicine, Sun Yat-sen University Cancer Center, Guangzhou, 510060 Guangdong P. R. China; 2 Nuffield Division of Clinical Laboratory Science, Radcliffe Department of Medicine, John Radcliffe Hospital, University of Oxford, Oxford, OX3 9DU UK

## Abstract

The close relationship between metastasis and establishment of tumor vasculature has inspired enormous research interests aiming to suppress metastasis via inhibiting the development of tumor vasculature. International experts gathered in Guangzhou, China on May 10–12, 2018 in The 4th International Meeting of Cancer and Blood Vessels to discuss the multiple ways for solid tumors to establish their vasculature. Vessel co-option is a mean by which a solid tumor takes advantage of the existing or newly induced blood vessels in the surrounding normal tissues to sustain tumor growth and metastasis. The underlying mechanisms of vessel co-option, the roles of pericyte, and the potential novel therapeutic targets have been discussed in the meeting.

## Text

Establishment of tumor vasculature has been closely linked to the spread of cancer cells, which is currently a main reason for treatment failure in many types of malignancies [1]. There are emerging evidences indicating that many types of solid tumors are able to acquire vasculature by several means. These include de novo angiogenesis with sprouting endothelial cells from peripheral pre-existing endothelial cells, vasculogenic mimicry with cancer cells directly forming the vascular channels by themselves, and vessel co-option of cancer cells that can hijack and exploit the surrounding normal vasculature for their own usage [2, 3]. Not until very recently, the important roles of vessel co-option in the development of metastatic lesion and treatment resistance have been reported by different research groups. In The 4th International Meeting of Cancer and Blood Vessels held in Guangzhou, China, on May 10–12, 2018, investigators from the United Kingdom, China, Spain, Sweden, Italy, the United States, and France presented their findings and raised in-depth discussions regarding the mechanisms of vessel co-option and other types of tumor vasculature establishment; with the aim of finding solutions to inhibit cancer progression. The exciting findings and thoughtful discussions of this meeting attracted over 150 researchers from multiple research fields.

Following the “angiogenesis delusion” [4], interest for anti-angiogenic therapy has considerably shrunken. However, at the same time, a number of studies, unravelling the complex relationship between cancer cells and blood vessels, have started to be published [5]. By now, the scientific community has agreed that there is more than just angiogenesis in the relationship between cancer cells and vessels: the discoveries of non-angiogenic tumours able to grow by exploiting pre-existing vessels, and of vascular mimicry are leading to new possible targets for therapeutics. Furthermore, other research groups are starting to find some genuine roles for antiangiogenic drugs in cancer treatment, although in a more limited and targeted way than before, they have accepted that there will be no “cure of cancer in 2 years” as contrarily declared by Jim Watson in 1998 [6]. This recent meeting in Guangzhou provided further evidences for cautious optimism.

The first clinical entities that could benefit from a more comprehensive understanding of how tumors establish their vasculature structures, are those of primary and metastatic brain tumours, since both have remarkably poor prognosis and sparse, if no treatments available at all. Particularly daunting is the dilemma of metastatic brain lesions for which no real treatment is yet available. Since both primary and metastatic brain tumours have been found to rely more frequently on vascular co-option rather than angiogenesis, the underlying molecular basis was among the most remarkable subjects discussed in the meeting. Molecules like Serpin, Stat3, laminins, and CDC42 are repeatedly found to be crucial for cancer cells to be able to co-opt blood vessels and evade cell death. This follows the suggestion that inhibiting these molecules, or others with similar functions, could be a realistic approach to the treatment of brain tumours. In addition, for clinical situations whereby patients bear high risks of developing secondary brain lesions, the most realistic possibility would be the development of therapeutic protocols designed for preventing the formation of those anticipated metastases rather than treating them when they become evident.

Vascular co-option can arise in different organs and each case can confer to different modalities. Lymph nodes are not estranged to this and are commonly the seat of co-option involving the high endothelial venules (HEVs). In sentinel lymph nodes, HEV exploitation leads to invariable vessel remodeling. The co-opted HEVs are not only critical for the growth of metastatic colonies, but also provide a gateway for the cancer cells to move into the bloodstream, thereby promoting distant metastases. This shows consistency with revelations from pioneering studies on melanoma that have identified co-option as being relevant to the cellular ability to spread throughout the body. A novel mechanism leading to tumour growth has been found in the liver where the molecular mechanism for the formation of sinusoidal vasculature in hepatocellular carcinoma have been linked to the expression of Ang2.

More focused works relating to classes of compound currently under scrutiny, e.g., tyrosine kinase inhibitors and therapeutic antibodies, as well as strategies for identification of new targets for inhibiting vascular co-option, were also presented and discussed. New developments offering innovative therapeutic ideas were linked to the accumulating evidences suggesting a tight interplay between angiogenesis and immunity. Hence, came the proposal of integrating anti-vascular drugs with immunotherapy, which could be a promising alternative to achieving better clinical outcomes.

The meeting also highlighted the prospects that targeting neo-angiogenesis can still be a useful addition to the present array of anti-cancer treatments. Besides, since compounds like kallistatin have demonstrated the ability to inhibit lymphangiogenesis in gastric cancer, this class of drugs could be promising synergists to control primary tumour growth and subsequent metastatic lesions and therefore suggest that it would be erroneous to ignore all-together the potential benefits of anti-angiogenic approaches.

Another emerging issue raised at the meeting was the role of pericytes. Because of their heterogeneity in origin, they have been found to play multiple roles in solid tumors, including sustaining blood vessel functions and promoting metastasis of cancer cells.

An interesting set of studies focused on the investigation concerning the biology of the non-angiogenic cancer cell and tried to answer these two main questions: (1) why some cancer cells failed to trigger angiogenesis and growth in this newly discovered fashion, and (2) what are the possible pathways for the driving force of this type of growth. Could they contain possible new therapeutic targets like as discussed in the conference, TRAP1? From these studies another interesting data set has emerged suggesting a possible switch from anaerobic to aerobic metabolism in non-angiogenic tumours. The possibility to target different aspects of the metabolic pathways supporting cancer cells is another emerging field of research [7] and its current status has been revived during the 2-day meeting. Finally, the importance of having more efficient mouse models for mechanistic and preclinical studies has been stressed and the recent innovations in this field have been additionally presented.

As a conclusive note, the findings suggesting that tumours can also grow in the absence of angiogenesis has now been confirmed and new data supporting this discovery keeps on appearing. Both the studies on the biology of these tumours and how the non-angiogenic cells interact with the pre-existing blood vessels and, in many cases, with the co-existing angiogenesis, is a new and emerging research field which seems promising for the forthcoming cancer treatment.
